# Supplementary material for: CD146+ Pericytes Subset Isolated from Human Micro-Fragmented Fat Tissue Display a Strong Interaction with Endothelial Cells: A Potential Cell Target for Therapeutic Angiogenesis
Source: Int J Mol Sci. 2022 May 22;23(10):5806. doi: 10.3390/ijms23105806 (PMC9144360; doi:10.3390/ijms23105806)
Supplement: Supplementary file 1 [file ijms-23-05806-s001.zip › ijms-1728370-supplementary.pdf]

Supplementary Data

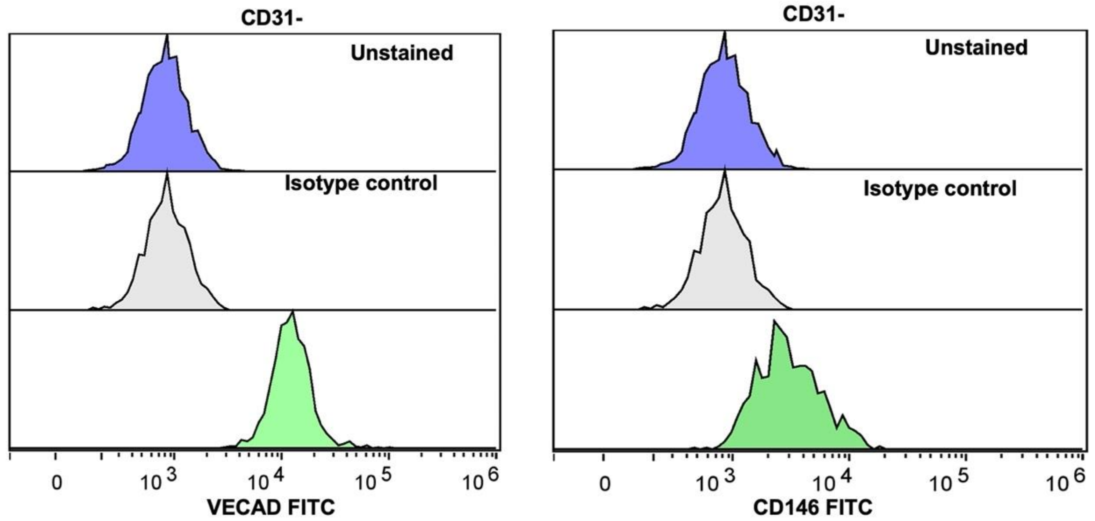

Figure S1. Flow cytometry histograms represent CD31⁻ PCs to be positive for VECAD (Vascular Endothelial Cadherin)-FITC. CD146-FITC was stained positive only for a small proportion of CD31⁻ cells. Histograms in the lowest panel represent the positive staining for specific antibodies as compared to the unstained and unrelated isotype-matched antibody in blue and light-grey histograms, respectively.

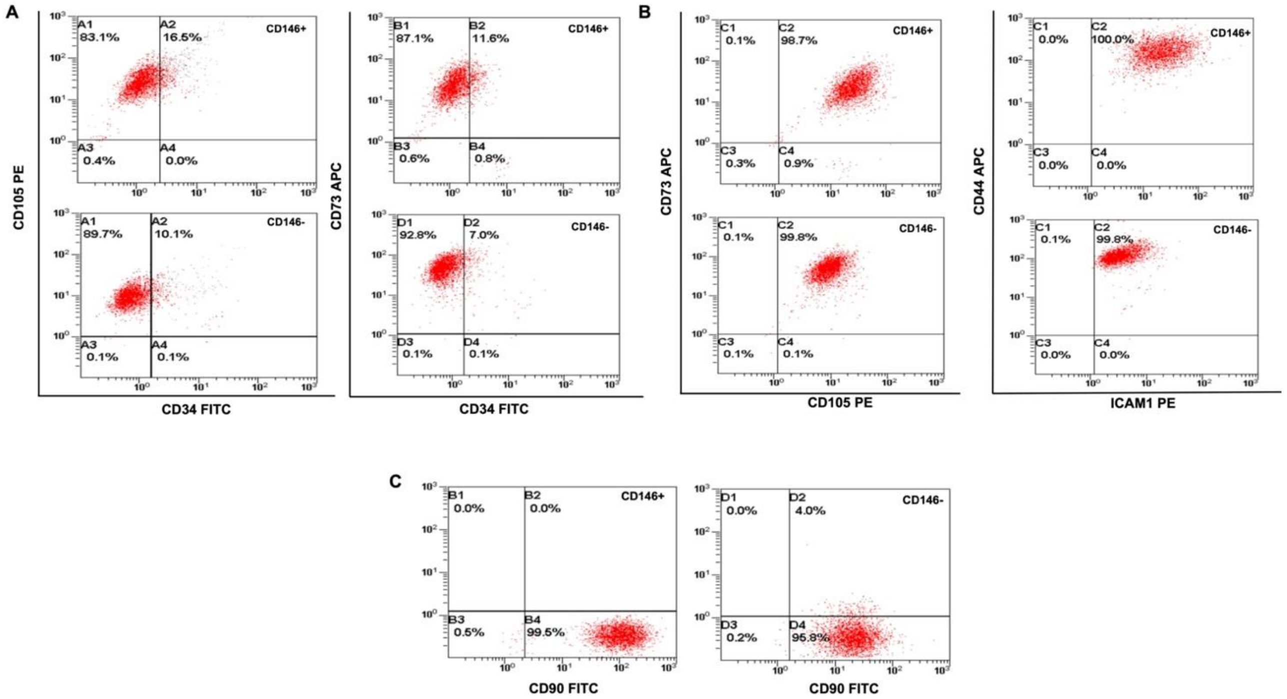

Figure S2. MSC marker analysis. (A, B) The dot plots represent the percentage of CD146$^+$ (upper side) and CD146$^-$ (lower side) cell population positive for typical MSC markers. **(C)** The dot plots represent the percentage of CD146$^+$ (left side) and CD146$^-$ (right side) cell population positive for CD90-FITC marker.

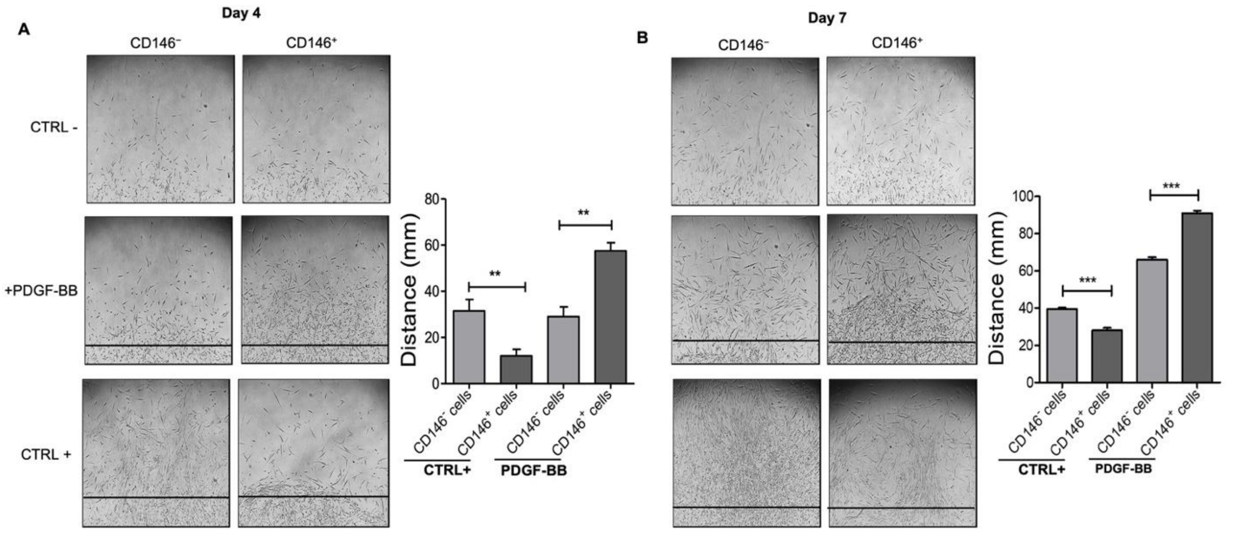

Figure S3. PDGF-BB promoted PCs migration for CD146$^+$ cells higher than CD146$^-$ cells evident at day 4 (A) until day 7 post-stimulation (B). On the other hand, CD146$^+$ cells were less migratory as compared to CD146$^-$ cells in the complete medium, used as a positive control, in the absence of PDGF-BB. The bar chart represents the relative distance migrated outwards the collagen layer towards the edge of the wells at day 4 (A) and day 7 post-stimulation (B). Images are representative of at least two independent experiments with similar results performed in triplicates. Statistical analysis was performed by one way-ANOVA and Bonferroni's post-test; *p $\leq$ 0.05, ** p < 0.01, ** *p <0.001. PDGF-BB–platelet-derived growth factor-BB, CTRL$^+$ — positive control; CTRL$^-$ — negative control.

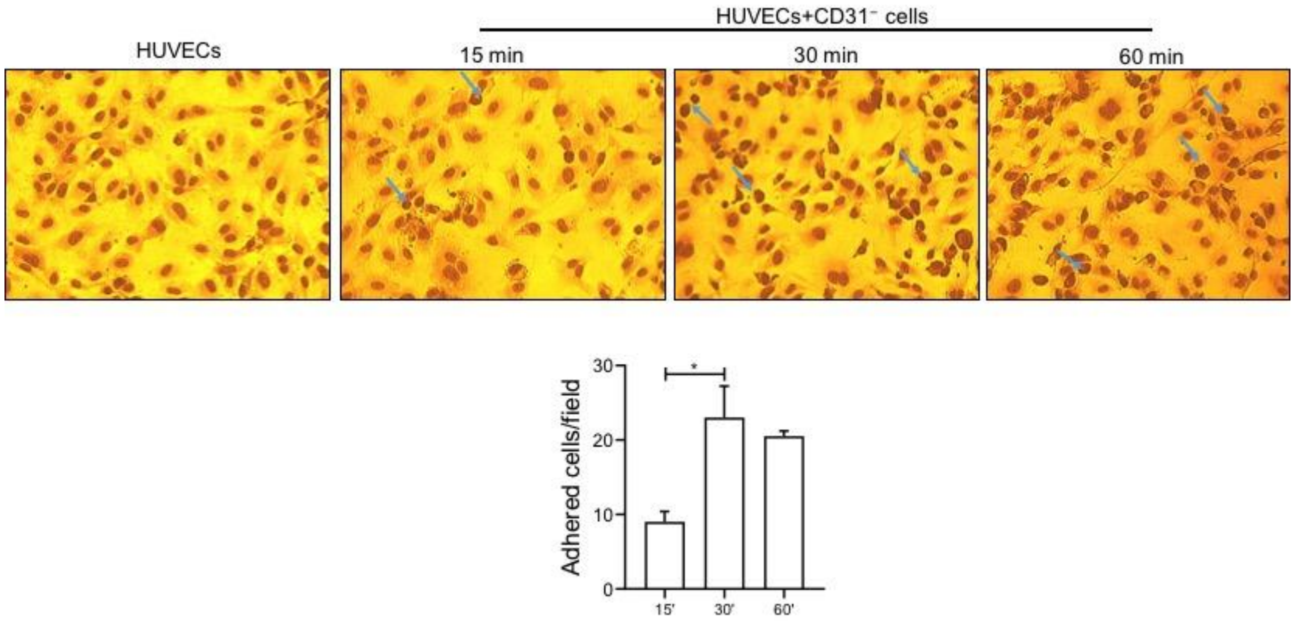

Figure S4. Adhesion Assay. CD31⁻ Pericytes detached from tissue culture flasks were seeded on cultured ECs monolayer. In the figure, adherent pericytes appear as darker round cells (for example, indicated by blue arrows) adhered to the elliptical-shape HUVECs monolayer. The adhesion of pericytes was observed over a time course of 15, 30, and 60 min. The left corner image represents HUVECs monolayer as a negative control in the absence of adhered pericytes. The bar graph displays the number of adhered cells per frame (counted for three different frames/well) for each condition performed in triplicates at 10X magnification. Statistical analysis was performed by one way-ANOVA following Bonferroni's post-test; * $p \leq 0.05$.

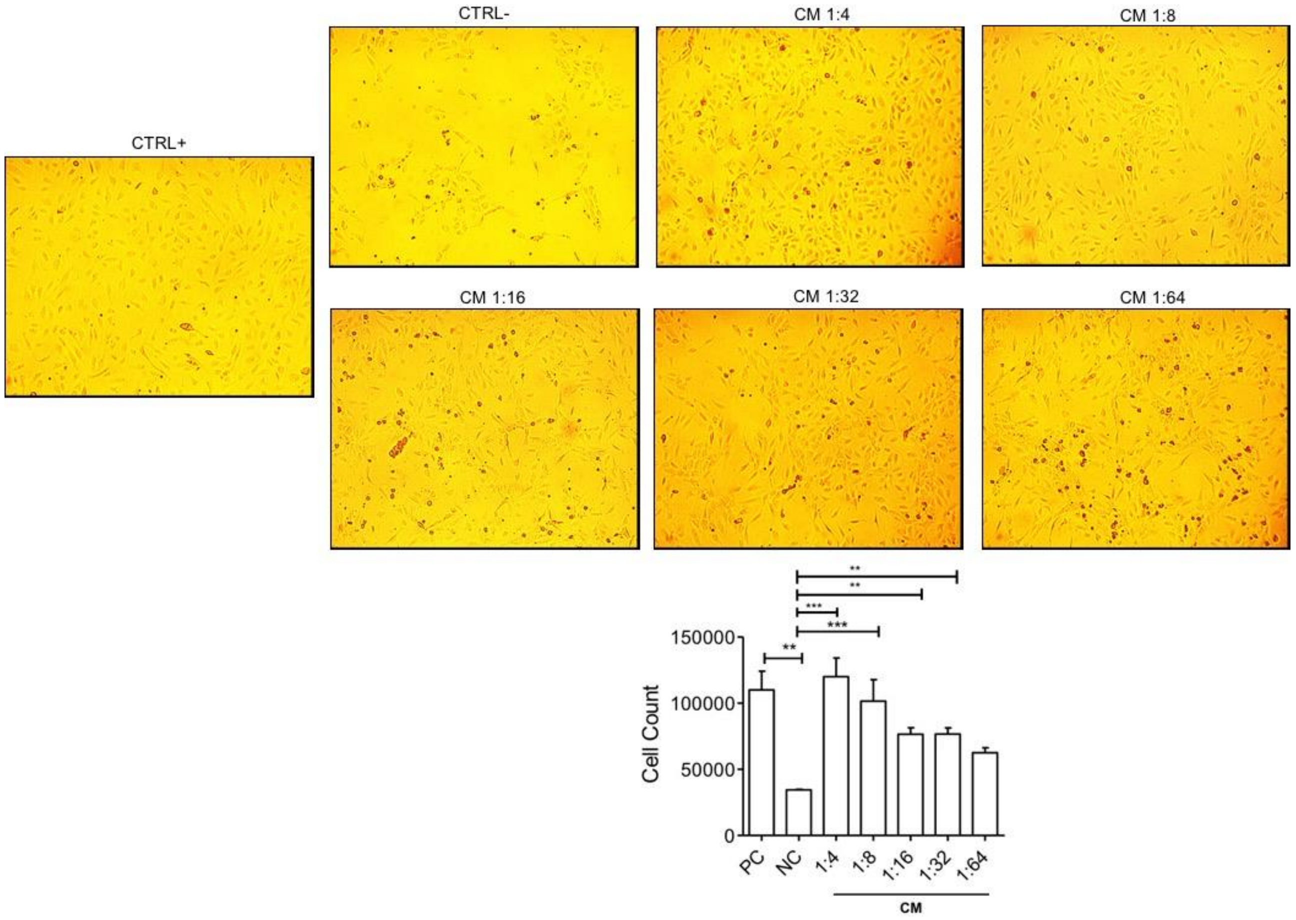

Figure S5. Cell Proliferation Assay. Cells cultured in basal medium and starvation conditions proliferated in the presence of conditioned medium (CM) from MFAT, serially diluted from 4-fold until 64-fold in RPMI medium. Images were taken after 16 h of treatment at 4X magnification. ECs cultured in the complete medium were used as a positive control. ECs cultured in starved conditions (EBM+0.5% FBS) were used as a negative control. The bar graph shows the cell count after overnight treatment with CM. Statistical analysis was performed by one way-ANOVA following Bonferroni's post-test; ** $p < 0.01$, ***$p < 0.001$.
